# Barriers and Attitudes of Primary Healthcare Physicians to Insulin Initiation and Intensification in Saudi Arabia

**DOI:** 10.3390/ijerph192416794

**Published:** 2022-12-14

**Authors:** Ali Jaber Alhagawy, Saeed Yafei, Abdulrahman Hummadi, Raed Abutaleb, Mohammed Hakamy, Turki Alzughbi, Nabeel Gharawi, Manal Moafa, Asma Mokali, Ibrahim Alhiqwy, Mousa Altherwi

**Affiliations:** 1 Jazan Endocrinology & Diabetes Center, Ministry of Health, Jazan 45142, Saudi Arabia; 2 Faculty of Medicine and Health Sciences, Taiz University, Taiz P.O. Box 6803, Yemen; 3 Family Medicine, Jazan Health Affairs, Ministry of Health, Jazan 45142, Saudi Arabia; 4 Faculty of Medicine, Jazan University, Jazan 45142, Saudi Arabia; 5 Nursing Department, Ministry of Health, Jazan 45142, Saudi Arabia

**Keywords:** clinical inertia, therapeutic inertia, insulin initiation, type 2 diabetes, primary healthcare physicians, barriers

## Abstract

Saudi Arabia is a country with high prevalence of diabetes, uncontrolled diabetes, and diabetes-related complications. Poor glycemic control is multifactorial and could be explained in part by physician and patient reluctance toward insulin or insulin inertia. This study aimed to address physician barriers toward insulin therapy in primary care settings. It included 288 physicians from 168 primary healthcare centers (PHC) in the Jazan region of Saudi Arabia. Participants responded to questionnaire investigating physicians’ attitude and barriers to insulin initiation and intensification in PHCs. In physician opinion, the most common barriers among their patients were fear of injection, lack of patient education, fear of hypoglycemia, and difficult administration. Physicians were reluctant to initiate insulin for T2D patients mostly due to patient non-adherence to blood sugar measurement, non-adherence to appointment or treatment, elderly patients, or due to patient refusal. Physicians’ fear of hypoglycemia, lack of staff for patient education, and lack of updated knowledge were the primary clinician-related barriers. Exaggerated fears of insulin side effects, patient non-adherence, limited staff for patient’s education, patient refusal, and inadequate consultation time were the main barriers to insulin acceptance and prescription.

## 1. Introduction

The global burden of type 2 diabetes (T2D) is steadily increasing with an estimated 537 million adults are living with diabetes in 2021 [1]. T2D is a progressive disease and its management involves different strategies that include diet management, exercise, glucose monitoring, anti-obesity measures, intake of oral antihyperglycemic agents, and, in some cases, insulin administration [2]. Worldwide, the prevalence of uncontrolled diabetes is still high despite improvement in treatment modalities and clear guidelines recommending treatment intensification [2]. Landmark trials have demonstrated that early achievement of glycemic targets in T2D leads to better outcomes and lower risks of microvascular and macrovascular complications [3].

Insulin is one of the major definitive effective treatments of diabetes and its effectiveness includes four critical accomplishments: initiation, adherence, persistence, and intensification [4]. Failure of healthcare providers to initiate, maintain, or intensify therapy when indicated is simply termed as clinical inertia [5]. Clinical or therapeutic inertia appears to be more pronounced when considering addition of insulin, particularly in insulin-naïve patients [5]. Reasons for this can be related to health care providers, diabetes patients, or the healthcare system [4].

Healthcare system barriers contribute to 20% of causes of therapeutic inertia such as availability of medications, cost, lack of resources, continuity of care, poor healthcare plans, high workload and time limitations, and ambiguity of roles in the primary care team [4,6]. Patient-related barriers represent about 30% of therapeutic inertia such as misconceptions regarding insulin risk, injection phobia, fear of weight gain, fear of hypoglycemia, negative impact on social life and job, poor health literacy, low self-efficacy and healthcare providers’ inadequacy [7,8].

Unfortunately, health care provider barriers make up 50% of the causes of therapeutic inertia and have been reported in both primary care physicians and specialists [6,9]. Healthcare provider barriers include lack of knowledge, training and experience, level of specialization, language barriers, fear of insulin-induced hypoglycemia or weight gain, perceived patient resistance resulting from fear of injections, beliefs about insulin non-effectiveness, and presumed patient non-adherence [4,10].

Saudi Arabia is a country with a high prevalence of diabetes and diabetes-related complications [11]. The prevalence of uncontrolled diabetes in Saudi Arabia is still high; only about 25% of T2D patients had good glycemic control [12,13]. Poor glycemic control is multifactorial and could be explained in part by physician and patient reluctance toward insulin or insulin inertia. There is great agreement that patterns of patient attitudes towards insulin closely resembled those held by physicians in the same country [10,14]. Therefore, addressing physician barriers is crucial to ensure that the necessary steps are taken to start tackling patient barriers. Different studies on Saudi diabetic patients reported that 24.4% to 34.6% of the Saudi diabetic patients refused or have negative attitudes toward insulin [15,16]. Few studies explored the barriers to insulin therapy among physicians in other regions of Saudi Arabia [17,18], however this problem needs more exploration from other regions of this country. The main objective was to address the barriers toward insulin initiation and intensification among primary care physicians (PCPs) so as to start tackling these barriers and improve quality of care and decrease the burden of diabetes-related complications.

## 2. Methods

### 2.1. Study Design and Sampling

This is an observational cross-sectional survey conducted between March and July 2022. Participants were restricted to registered physicians and family medicine specialists providing healthcare at the 168 PHCs in the Jazan region of Saudi Arabia. A questionnaire was emailed to all PCPs registered for Jazan PHC administration with an invitation to participate. Physicians received two reminders to complete the online survey.

### 2.2. Study Questionnaire

A structured questionnaire was developed after a comprehensive review of the literature investigating physicians’ attitude and barriers to insulin initiation and intensification worldwide with some adaptation to ensure compatibility with the Saudi primary healthcare sector [19,20,21,22]. The questionnaire consisted of two parts and was reviewed for content validity by an expert group of family physicians and diabetologists.

The first part of the questionnaire included demographic data including age, gender, nationality, specialty training, and experience duration. Other information related to T2D patients included number of T2D patients managed per week, number of new cases per month, percent of patients on insulin, availability of different types of insulin and delivery systems in the PHCs, and availability of trained educators, dietitian and social workers in the PHCs.

The second part of the questionnaire included questions related to barriers and attitudes to insulin prescription. Three questions with multiple items focused on the barriers towards initiation and intensification of insulin therapy. Items were developed depending on an extensive review of similar studies [19,20,21,22]. The first question included twelve items related to the commonly reported barriers to insulin acceptance among T2D patients. The second question included seven items about the patient-related factors that make PCPs reluctant to prescribe insulin. The third question included seven items assessing the physician-related factors that make them reluctant to initiate or intensify insulin. In addition, two closed-ended questions asking if the physician was confident to initiate insulin therapy, or if they prefer to delay insulin until it becomes absolutely necessary.

### 2.3. Statistical Analysis

Data entry and analysis were performed using the Statistical Package for the Social Sciences software. Descriptive analyses were performed. Responses to most questions were represented as absolute or relative frequencies. Means and standard deviations (SD) were calculated for continuous variables where applicable. The Chi-square test was used to compare practitioner and specialist response.

## 3. Results

### 3.1. Physicians Characteristics

We invited 463 primary PCPs from the 168 PHCs in Jazan; 288 of them completed the questionnaire, with a response rate of 62.2%. The mean age of the respondent was 38 ± 7.7 years (range 25–60 years). Most of the respondents (58.3%) were men (168/288). Eighty-four participants were of Saudi nationality representing 29.2% of the respondent physicians. The sample included 202 general practitioners (70.1%) and 86 participants had completed higher academic training in family medicine and were licensed as specialists. Most of the physicians (71.5%) had been in practice for more than 5 years and 73.3% reported seeing at least 10 patients a week. Diabetes educators and dietitians were available for 22.9% and 20.8% of the PCPs, respectively, while social workers were available only for 11.8%.

### 3.2. Insulin in the Daily Practice of PCPs

About 40.3% of the PCPs reported that more than 25% of their patients were on insulin. Approximately 85.4% of the physicians admitted that they were confident to initiate insulin therapy by themselves. When asked about the type of insulin and insulin devices available in the PHCs, 100% of the PCPs had access to insulin pens and only 30% of PCPs had access to insulin syringes; they rarely prescribe this type of insulin in the PHCs. All kinds of insulin are available to PCPs, except for Degludec and Glulisine insulin.

PCPs selected these criteria for insulin initiation: HbA1c levels (88.9%), if the patient was on maximum doses of oral antihyperglycemic medications with an HbA1c > 7% (62.5%), and if fasting blood sugar was more than 250mg/dl (45.8%). Other less commonly selected criteria included: patient preference (27.8%), the potential side effects of insulin as compared with those of other antihyperglycemic medications (25%), patient weight (22.2%), the patient work schedule and lifestyle factors (16%), availability of nurses, diabetes educators and others to implement and follow the insulin treatment (16.7%), and the cost of insulin (9.3%).

According to their answers, PCPs could intensify insulin depending on HbA1c levels (89.9%), patient compliance with the prescribed insulin (52.8%), and patient willingness to increase insulin doses (36.6%). Other less selected criteria included the risk of hypoglycemia, the patient’s work schedule and lifestyle factors, the availability of nurses, diabetes educators, and others to follow the insulin treatment, and the cost of insulin.

### 3.3. Barriers to Insulin Initiation and Intensification

For the first question, PCPs were asked about the biggest barriers for their patients towards initiation and or intensification of insulin therapy (Table 1). This question included twelve items, so physicians were asked to give their response to every item. In physician opinion, the most popular perceived barriers among their patients were fear of injection (81.1%), lack of patient education about diabetes and its complication (68.6%), fear of hypoglycemia (50.7%), and technical difficulty involved in administration (50.7%). Other barriers to insulin acceptance among T2D patients in PCPs opinion included patients concern about personal failure in controlling diabetes, worries about embarrassment and social stigma, fear of weight gain, fear of death, religious/cultural beliefs, job instability, decreased life span, and insulin cost.

### 3.4. Reasons for Reluctance to Initiate Insulin

According to their responses, 14.6% of the physicians admitted that they were not confident enough to initiate insulin therapy. A further 28.1% of the PCPs prefer to delay insulin therapy until it becomes absolutely necessary. In response to the question asking about the patient-related factors that make PCPs reluctant to prescribe insulin, physicians were reluctant to initiate insulin for T2D patients mostly due to patient non-adherence to self-monitoring of blood glucose (SMBG) (73.6%), non-adherence to appointment or treatment (52.1%), old patients due to fear of hypoglycemia (51.4%), or due to patient refusal (41.7%). Low socioeconomic level, body weight, and presence of cardiovascular disease were minor factors considered by PCPs (Table 2).

In response to the question asking about the physician-related factors that make them reluctant to initiate or intensify insulin, different factors were selected. Insufficient training and experience reported by 25.7%, and lack of updated knowledge about insulin initiation and intensification was reported by 17% of the PCPs. However, about half of them admitted that fear of insulin-induced hypoglycemia (46.5%) and lack of staff for patient training and education (49.3%) were the main barriers that made them reluctant to prescribe insulin for T2D patients. The list of barriers also included lack of time for patient education, fear of weight gain, and unavailability of HbA1c for insulin intensification (Table 2).

Of the PCPs, 28.9% reported that the education given to their patients was not adequate to allow insulin initiation. Furthermore, 37.1% of respondents felt that the consultation times are not sufficient to initiate insulin therapy while 32.1% felt that appointment times were not sufficient to review insulin initiation.

## 4. Discussion

This study was conducted on PCPs in Jazan region of Saudi Arabia. The main objective was to address physician barriers toward insulin therapy so as to start tackling these barriers and improve the quality of care and decrease the burden of diabetes-related complications.

In the present study, most of the PCPs were confident to initiate insulin and most of them prescribe insulin in their daily practice. Saudi Arabia has already started shifting focus and investment from secondary and tertiary healthcare facilities toward primary healthcare, where most T2D patients are treated in primary care centers [23]. This shifting strategy, combined with the obligatory continuous medical education required from every healthcare worker, and popularity and easy access to educational material and online courses on diabetes management, could explain the high rate of confidence and experience on insulin therapy among PCPs.

Most of the PCPs in this study have been working in primary healthcare for at least five years and provide care for at least 10 diabetes patients per week. More than one third (40.3%) of the physicians claimed that approximately 25% of their patients are on insulin. Additionally, insulin was available for every physician, free of charge, and insulin pens are available for almost all of the physicians: we are dealing with physicians who are working in an area with a high prevalence of diabetes, who are suggested to have average experience with diabetes management.

There is a great consensus that the final goals of diabetes management are prevention of long-term complications and improvement of quality of life, which can be achieved only with maintenance of glycemic control over time. Unfortunately, this is a complex task as diabetes is a progressive disease that requires the timely optimization of treatment, leading in majority of cases to insulin therapy [24,25]. However, in different clinical practice, the use of insulin tends to be delayed and irreversible complications can already be present by the time it is started. This delay is multifactorial, and often it is a physician rather than the patient who decides to postpone insulin therapy [22].

Most of the PCPs in this study (88.9%) agree that insulin therapy should be initiated depending on the degree of hyperglycemia or HbA1c levels, especially if the patient was on a maximum dose of oral antihyperglycemic medications with an HbA1c > 7%. This agreement coincides with ADA/EASD guidelines which recommend that patients on dual or triple therapy where HbA1c is above the individual target, or in those with extreme and symptomatic hyperglycemia or HbA1c > 10, insulin therapy should be initiated [2].

Regarding barriers to insulin initiation, most of the PCPs admitted that their patients were resistant and unwilling to begin insulin mainly due to fear of injections, lack of education about diabetes, fear of hypoglycemia, and difficult administration technique in addition to others. Furthermore, physicians in this study are reluctant to initiate insulin mainly due to patient- and system-related rather than physician-related factors. In physician opinion, non-adherence to SMBG, non-adherence to appointment or treatment, old patients with high risk of hypoglycemia, patient refusal, and lack of education staff were the main reasons for reluctance. The minor factors reported by PCPs are lack of updated knowledge or experience, lack of time for patient education, low socioeconomic level, body weight, and presence of cardiovascular disease.

The perceived barriers to insulin initiation in this study mirrored some of the patient-reported barriers in Saudi Arabia. Batis et al. [15] found that the most common barriers to insulin in Saudi T2D patients are fear of needle injections, fear of hypoglycemia, weight gain, difficult administration technique, and social stigma or embarrassment. Other barriers reported by PCPs were also reported in similar surveys on T2D patients from Saudi Arabia [15,26] including fear of weight gain, fear of job instability, and worry about impaired quality of life.

However, while physicians in this study think that most of their patients fear needle injections (80.5%), studies on diabetes patients reveled that fear of needle injections was not the most common reason for their refusal to initiate insulin [26,27]. Fear of needle injections is a significant worldwide problem among T2D patients and is often related to insufficient knowledge and past experience. Unfortunately, physicians may overestimate this fear of injection as a cause of patient resistance to insulin initiation [7,14]. This represents areas for education for patients and PCPs aiming to enhance good communication between them and applying effective strategies to explore and resolve these fears [4].

The occurrence of hypoglycemia with any diabetes therapy is a serious concern among patients and physicians. Different surveys on patients and physicians found that hypoglycemia related to insulin therapy is the main obstacle that delays insulin initiation and intensification in patients with T2D [10,19]. In this study, most of the PCPs (73.6%) reported being unwilling to initiate insulin for patients who do not adhere or are unable to self-monitor their blood sugar, or for those at high risk of hypoglycemia (e.g., the elderly). Furthermore, they believe that most of their patients could refuse insulin, and about half of the PCPs (46.5%) reported delaying insulin due to their own fear of hypoglycemia. This concern of hypoglycemia is commonly shared among different studies including physicians from Saudi Arabia [18] and other countries [20]. However, other studies found that hypoglycemia is of less concern among physicians in other countries. Kim et al. [28] reported that physicians delayed insulin therapy mostly because of patient refusal or because of concern about the patient’s compliance (26.5%) rather than concerns about hypoglycemia or weight gain.

There are limited studies on the incidence of hypoglycemia in Saudi diabetes patients. One study [29] reported a rate of 12.5% among T2D that mainly related to delayed or omitting meals after insulin injection. Another study reported a higher incidence during the daytime fasting in Ramadan [30]. These have great clinical implications as physicians should be aware of the time and risk factors of hypoglycemia and their exaggerated fears and perceptions mean delaying insulin initiation and, subsequently, more diabetes-related complications.

Undoubtedly, knowledge, training, and experience of the physicians are important factors affecting their decisions especially when dealing with insulin initiation and intensification. In our study, only 14.6% of PCPs admitted that they were not confident enough to initiate insulin therapy. About 25.7% of physicians admitted that insufficient training and experience and lack of updated knowledge (17%) were barriers to initiating insulin. Different surveys from other regions of Saudi Arabia reported higher rates of low confidence, less experience, and insufficient knowledge among PCPs [17,18]. Surveys from other countries highlighted the gaps in physician education and recommended targeted medical education programs to improve physician knowledge, attitudes, and beliefs toward insulin [19,31].

In this study, 27.8% of the PCPs admitted that the education given to patients was insufficient to allow the initiation of insulin therapy. Moreover, most of the PCPs (68.8%) believe that lack of education about diabetes and its complications among patients is another barrier among patients to accept insulin. Lack of knowledge on diabetes and insulin among diabetes patients was reported as a barrier to insulin prescription in different studies [32].

Lack of staff and time for patient education are common causes for delayed insulin treatment and for low education among diabetes patients [32]. Time limitations with long appointment periods and short consultation time were commonly cited by both PCPs and patients in different studies [9,20,33]. The strategy of using videos explaining pen injection and titration technique could take short time and may allay fears regarding the complexity of treatment [9].

Stigmatization of diabetes can lead to negative psychological and clinical outcomes. In the current study, 16.7% of the PCPs admitted that their patients refuse insulin use due to fear of stigma or discrimination. Diabetes stigma was frequently cited as a barrier to insulin among diabetes patients in different communities [6,14,15,32]. One study from Saudi Arabia [26] found that 24.7% of T2D patients have negative attitudes to insulin due to fear of social stigma.

Availability of HbA1c result was another barrier selected by 24.3% of PCPs, so they cannot make important decisions regarding insulin initiation or intensification, especially in patients who have difficulty with SMBG. According to the Ministry of Health’s diabetes management policy, point-of-care HbA1c testing should be available in each PHC and every patient with diabetes should be provided with a glucometer and glucose measurement strips. However, these glucometers may be damaged or lost and strips shortage is common and, in some occasions, HbA1c tests may be not available. These issues need to be resolved in future policies, increasing access to HbA1c testing, which is a cost-effective strategy in reducing clinical inertia and improving glycemic control [34].

The results of this study indicate that there are different barriers related to physicians, their patients, or the healthcare systems. Dealing with these barriers among physicians and patients and adopting suitable strategies to resolve these barriers is of great clinical importance to improve glycemic control and reduce the burden of diabetes complications. In this context, the telemedicine program which was adopted recently in Saudi Arabia is an excellent strategy, aiming to improve patient communication with physicians and other healthcare workers. The decentralization of diabetes management from secondary and tertiary care to primary healthcare is another cost-effective strategy adopted in Saudi Arabia.

This study has some limitations. It does not include patients or healthcare providers other than physicians, so the results could give a broader idea about the barriers among these groups. Although quantitative studies enable a larger sample, qualitative or a mixed-method design could be more appropriate to explore barriers that might be not have been reported previously or that are unique to a specific community. Another limitation is that this study included PCPs from one region of Saudi Arabia, but it included comparison with similar studies from other regions. Furthermore, this study is not a longitudinal study linking glycemic control to insulin initiation or inertia to clearly explore the problem, which is an interesting point for future research.

## 5. Conclusions

In conclusion, this survey of PCPs highlighted multiple barriers for insulin therapy. Limited staff for diabetes education, low patient education, patient refusal, exaggerated fears of insulin side effects, and inadequate consultation time were the main barriers to insulin acceptance and prescription. Future studies comparing patient and physician barriers and application of practical strategies to improve patient and physician knowledge and resolve these barriers are warranted.

## Figures and Tables

**Table 1:** Primary care physician perceived barriers to insulin therapy.

For Most of My Patients, These Are the Biggest Barrier (s) to Their Acceptance of Insulin Therapy	Total *n* = 288	Practitioner *n* = 202	Specialist *n* = 86	*p*
Fear of injection	80.6	79.2	83.7	0.376
Lack of education about diabetes and insulin	68.8	69.3	67.4	0.755
Fear of hypoglycemia	50.7	48.5	55.8	0.257
Technical difficulty in insulin administration	50.7	46.5	60.5	0.030 *
Concern about personal failure in controlling diabetes	26.4	18.8	44.2	<0.001 *
Embarrassment/Social Stigma	16.7	14.9	20.9	0.205
Fear of weight gain	14.6	12.9	18.6	0.207
Religious/Cultural Beliefs	11.1	11.9	9.3	0.524
Fear of death	10.4	8.9	14	0.200
Worry about job instability	8.3	5.9	14	0.024 *
Decreased Life Span	6.3	2.0	16.3	<0.001 *
Insulin cost	4.9	4	7	0.276

Data are presented as percent, * *p* is significant at the 0.05 level.

**Table 2:** Reasons for reluctance to initiate insulin.

**I May Be Reluctant to Initiate Insulin Therapy for My Patients with T2DM Who**	**Total** ***n* = 288**	**Practitioner** ***n* = 202**	**Specialist** ***n* = 86**	***p***
Are reluctant to start it	41.7	43.6	37.2	0.317
Do not adhere to their appointments and treatment regimen	52.1	48.5	60.5	0.063
Do not adhere to their self-monitoring of blood sugar	73.6	73.3	74.4	0.839
Are from a low socioeconomic level (poor patient’s cognitive abilities)	28.5	24.8	37.2	0.032 *
Are ≥75 years of age because of the risk of hypoglycemia	51.4	50.5	53.5	0.642
Have excess weight (BMI ≥ 35) because of the risk for weight gain	24.3	29.7	11.6	0.001 *
Have cardiovascular diseases	13.9	13.9	14	0.983
**I may be reluctant to initiate insulin therapy for my patients with T2DM because**				
I do not follow the medical updates on insulin therapy	17	17.8	15.1	0.567
I do not have enough experience with insulin therapy	25.7	30.2	12.8	0.002 *
I do not have enough time for patient education and training	28.8	29.7	26.7	0.612
I do not have enough staff for patient education and training	49.3	41.6	67.4	<0.001 *
I worry about the risks of hypoglycemia	46.5	45.5	48.8	0.608
I worry about the risks of weight gain	13.2	12.9	14	0.804
HbA1c is not available for me to guide insulin therapy	24.8	22.4	23.9	0.638

Data are presented as percent, * *p* is significant at the 0.05 level.

## Data Availability

The data presented in this study are available on request from the corresponding author.
